# Selective adipogenic differentiation of human periodontal ligament stem cells stimulated with high doses of glucose

**DOI:** 10.1371/journal.pone.0199603

**Published:** 2018-07-06

**Authors:** Chao Deng, Yi Sun, Hai Liu, Wei Wang, Jingmen Wang, Feimin Zhang

**Affiliations:** 1 Jiangsu Key Laboratory of Oral Diseases, Nanjing Medical University, Nanjing, China; 2 Department of Prosthodontics, Affiliated Hospital of Stomatology, Nanjing Medical University,Nanjing, China; 3 School of Stomatology, Wannan Medical College, Wuhu, China; Università degli Studi della Campania, ITALY

## Abstract

Periodontal tissue damage, accompanied by the degradation and destruction of periodontal tissue collagen, is one of the most clinically common complications and difficulty self-repair in patients with diabetes. Human periodontal ligament stem cells (PDLSC) are the undifferentiated mesenchymal cells that persist in the periodontal ligament after development of periodontal tissue and the ability of PDLSC osteogenic differentiation is responsible for repairing periodontal tissue defects. However, the reasons of high glucose environment in diabetic patients inhibiting PDLSC to repair periodontal tissues are unclear. To address these issues, we propose exposing PDLSC to high-sugar mimics the diabetic environment and investigating the activity of osteogenic differentiation and adipogenic differentiation of PDLSC. At the cellular level, high glucose can promote the adipogenic differentiation and inhibit osteogenic differentiation to decrease the self-repair ability of PDLSC in periodontal tissues. Mechanistically at the molecular level, these effects are elicited via regulating the mRNA and protein expression of C/EBPβ, PPAR-γ.

## Introduction

Diabetes is a metabolic disorder characterized by hyperglycemia, its complications involving many organs such as cardiovascular, eye, kidney and foot, to name a few[1, 2]. Studies have shown that the major diabetic microangiopathies such as diabetic retinopathy eventually lead to the loss or even loss of vision[3]. Diabetes can lead to skin wound healing delay or gangrene, leading to diabetic foot disease[4].

Periodontal tissue damage is one of the most clinically common complications in patients with diabetes[5]. The relationship of pathogenesis in diabetes and periodontal tissue damage is similar, both are multifactorial diseases[6]. It is well known that diabetes itself does not cause periodontitis[7]. However, due to diabetes can cause glucose metabolism disorder, microangiopathy, end products of glucose-induced endings, and tissue healing ability, resulting in periodontal microcirculation, eventually lead to periodontal tissue damage[8].

Human periodontal ligament stem cells (PDLSC) are undifferentiated mesenchymal cells that persist in the periodontal ligament after periodontal tissue development[9, 10]. Similar to bone marrow stromal stem cells (BMSCs), adipose-derived stem cells (ASCs)[11, 12], PDLSC not only have the ability of self-renewal, but also have the potential to differentiate into fat, cartilage, nerve and muscle cells under certain inducing conditions. Study has shown that changes in the biological activity of PDLSC are responsible for the periodontal tissue damage, and its osteogenic ability can repair defects in periodontal tissue[13]. At the same time, PDLSC is also one of the seed cells for the treatment of periodontal tissue damage[14].

Studies have shown that high glucose microenvironment has an effect on adipocyte differentiation in stem cells[15, 16]. High glucose decreased adipocyte differentiation and promoted adipogenic differentiation of BMSCs[17, 18]. There is still controversy, the concentration of glucose at 25 mM generally inhibiting adipogenic differentiation of 3T3-L1 cells, but other studies have shown no positive effect[19]. Some studies have also investigated the effect of high glucose on the process of osteogenic differentiation in PDLSC, but the high glucose on the periodontal differentiation related information is rare[20, 21]. Currently, there is not sufficient evidence to explain the reason for the difficulty of periodontal repair in diabetic patients.

Herein, first of all, PDLSC was cloned and cultured by tissue block method and limiting dilution method *in vitro*. Then, PDLSC was exposed to high glucose, and the formation of lipid droplets was observed by oil red O staining. Finally, the gene and protein expressions of CCAAT-enhancer-binding protein β (C/EBP β) and peroxisome proliferator- activated receptor γ (PPAR-γ) were determined using RT-PCR and western blot.

## Materials and methods

### Ethical considerations

Ethical approval was obtained from Wannan Medical College of Medicine Institutional Review Board. Permission to carry out the study was obtained from Wuhu City, Anhui Province Council Authority Education office and the respective heads of schools.Written consent was obtained from the parents/guardians on behalf of the children and assent was also obtained from the study children’s teeth in accordance with Helsinki Declaration.

### Isolation, culture and purification of PDLSC

As previously described[22–25], freshly extracted permanent teeth that needed to be removed for orthodontic reasons but were otherwise healthy, were collected from approximately 16-year-old subjects, washed repeatedly with sterile phosphate-buffered saline (PBS; Boster Wuhan, China) and the middle 1/3 of the root periodontal ligament tissue was removed. Single cell-derived colony cultures were obtained using the limiting dilution technique as previously described[26]. All samples were collected at the first affiliated hospital of Wannan Medical College. And written informed consent was obtained from all subjects.

### Experimental groups

Third-generation cells were randomly assigned to two groups: a normal control group (N-PDLSC), which was cultured in a low-glucose environment (DMEM medium comprising 5 mmol/L glucose) to simulate the normal blood glucose levels, and the experimental group (T-PDLSC), which was cultured under an elevated glucose concentration (DMEM medium comprising 25 mmol/L glucose). It was reported that more than 8 mmol/L of sugar concentration was an uncontrolled diabetes blood glucose concentration in many literature, so many studies had selected the different sugar concentration to simulate the high glucose microenvironment, such as 12 mmol/L, 16.5 mmol/L, 25 mmol/L, 35 mmol/L and 40 mmol/L. In our paper, the sugar concentration of 25mmol/l was chosen as the high sugar concentration to be studied. Cells from both groups underwent induced osteogenic and adipogenic differentiation.

### *In vitro* induction of PDLSC osteogenesis and adipogenesis

As previously described[27], third-generation PDLSC were seeded into 6-well plates at a density of 1 × 10^5^ cells/mL, to which L-DMEM medium comprising 5% FBS was added, and the cells cultured at 37°C in an atmosphere comprising 5% CO_2_. After the cells proliferated to 70% confluence, they were separated into groups and transferred into osteogenic (100 nM dexamethasone, 50 μg/ml of ascorbic acid and 5 mM β-glycerophosphate; Sigma, USA) and adipogenic (0.5 mM methylisobutylxanthine, 0.5 mM hydrocortisone, and 60 mM indomethacin; Sigma, USA) induction media, respectively, with media changes every 3 days. After 21 days of differentiation induction, each group was separately stained using alizarin red (Sigma, USA) for osteogenic differentiation, and oil red O (Sigma, USA) for adipogenic differentiation.

### Staining and quantification of lipid droplets and calcified nodules

As previously described[28], samples comprising cells that underwent adipogenic and osteogenic induction for 21 days. The cells were fixed with 75% ethanol and washed three times with PBS. Then the cells were stained with Oil red O (Sigma) solution or 2% Alizarin red S (Sigma) for 30 min, respectively. The cells were washed 3 times with PBS for 5 min each time. Finally, the Alizarin red treatment group was replaced with a 5% SDS solution in 0.5 N HCl for 30 min and absorbance was measured at 405 nm (Bio-Tek Instruments, Winooski, VT, USA). ALP staining was determined by 5-bromo-4-chloro-3-indolyl phosphate according to the manufacturer’s suggested protocol.

### Flow cytometry for the determination of surface molecules of the periodontal ligament cells

For screening of the cultured human PDLSC, the third-generation PDLSC were seeded into 6-well plates at a density of 1 × 10^5^ cells/mL, and the cells cultured at 37°C in an atmosphere comprising 5% CO_2_. After the cells proliferated to 70% confluence, the culture medium was discarded, and the cells were washed with sterile PBS two times, after which they were treated with 2mL of a 3 mg/L trypsin solution for 1 min, after which the reaction was neutralized with culture medium. The resulting neutralized cell suspension was centrifuged for 5 min, the supernatant discarded, and the cells resuspended in PBS comprising 3% FBS, yielding a single-cell suspension of periodontal ligament cells. Subsequently, the cell density was adjusted to 3.0 × 10^9^/L, 200 μL aliquots of the resulting cell suspension were transferred into individual 1.5 mL polypropylene tubes, and 2 μL aliquots of solutions comprising fluorescently-labelled mouse monoclonal antibodies (1:100 working dilution; see reagents section) reactive against CD29, CD90, CD105, CD14, CD31, CD45. (San Diego, CA, USA), CD146, stro-1(Minneapolis, MN, USA) and respectively, were individually added to separate tubes at room temperature in the dark, and the resulting mixtures incubated in the dark in a refrigerator at 4°C for 1h. Subsequently, the cells were washed with PBS comprising 3% FBS two to three times. Background labelling was determined using the same monoclonal antibodies. The thus labelled fluorescent cells were analyzed using a flow cytometer (Cytomics^TM^ FC 500 MCL/MPL, Beckman Coulter, USA), and the rate of cells positive for each of the indicated antigens was calculated using the flow cytometer’s own software, and expressed using percentage points (%) as units.

### Quantitative real-time polymerase chain reaction

Quantitative real-time polymerase chain reaction (qRT-PCR) was used to measure the expression of C/EBP β and PPAR-γ during adipogenic differentiation and the expression of ALP, osteopontin (OPN), RUNX-2 in the cells from both groups following osteogenic differentiation, as well as the expression of TCF1, TCF3, TCF4, LEF and LPL gene in the process of osteogenesis. After adipogenic and osteogenic induction of periodontal ligament cells from both groups for 7 d, the cells were collected and the total RNA extracted by using TRLzol (Invitrogen). qRT-PCR was performed using Applied Biosystems 7500 Real-time PCR Detection System (Applied Biosystems,Foster City, CA, USA). The β-actin gene was used as internal control. The primer sequences used in this study were listed in Table 1. The qRT-PCR reactions were conducted in a 20μL setup, with three wells for each replicate and three replicates for each group.

**Table 1:** Primer sequences used in real-time qRT-PCR.

Gene	Forward primer	Reverse primer
***PPAR-γ***	5’ CGAGAAGGAGAAGCTGTTGG3’	5’TCAGCGGGAAGGACTTTATG3’
***C /EBPβ***	5’CACAGCGACGAGTACAAGA3’	5’AGCTGCTCCACCTTCTTCTG3’
***Runx-2***	5’CACTGGCGCTGCAACAAGA3’	5’CATTCCGGAGCTCAGCAGAATAA3’
***OPN***	5’GCCGACCAAGGAAAACTCACT3’	5’GGCACAGGTGATGCCTAGGA3’
***ALP***	5’GGACCATTCCCACGTCTTCAC3’	5’CCTTGTAGCCAGGCCCATTG3’
***TCF1***	5’ CTACCCTGGGATTCAGGAAA 3’	5’ ACCGCATTTCTCCTTGACTT 3’
***TCF3***	5’ GCTGTGGAAACCTGGCTTAT 3’	5’ AAGGCAGCATCCTTGCTAAT 3’
***TCF4***	5’ GCTCCACCTCAAGAGTGACA 3’	5’ GAGGCTCTGAGGACACCTTC3’
***LEF1***	5’CGAAGAGGAAGGCGATTTAG 3’	5’ TCCTGAGAGGTTTGTGCTTG3’
***β-actin***	5’GGGAAATCGTGCGTGACATTAAGG3’	5’CAGGAAGGAAGGCTGGAAGAGTG3’

### Western blot analysis

Western blotting was used to determine the PPAR-γ, Runx-2, C/EBPβ, ALP, LPL, adiponectin,osteocalcin (OCN) and OPNprotein expression[29]. After adipogenic and osteogenic induction of periodontal ligament cells from both groups for 7 d, the cells were collected and the total proteins extracted using lysis buffer (10 mM Tris-HCl, 1 mM EDTA, 1% SDS, 1% Nonidet P-40, 1:100 proteinase inhibitor cocktail, 50 mM β-glycerophos-phate, 50 mM sodium fluoride). After sodium dodecyl sulfate-polyacrylamide gel electrophoresis(SDS-PAGE) on an 8% polyacrylamide gels, the proteins were transferred to a PVDF membrane (Millipore, Billerica, MA, USA). After which the membrane was blocked with 5% skim milk for 2h, the primary antibody was contacted with the membrane at a working dilution and PPAR-γ (Abcam, Cambridge, UK) finally detected via chemiluminescence (Amersham Biosciences, Piscataway, NJ, USA).

### Statistical analysis

SPSS17 software (SPSS, San Rafael, CA) was used for statistical analysis. The means of two independent samples were compared using Student’s t-test, and differences were considered statistically significant at p<0.05.

## Results

### PDLSC isolation, purification and subculture

**Fig 1:**
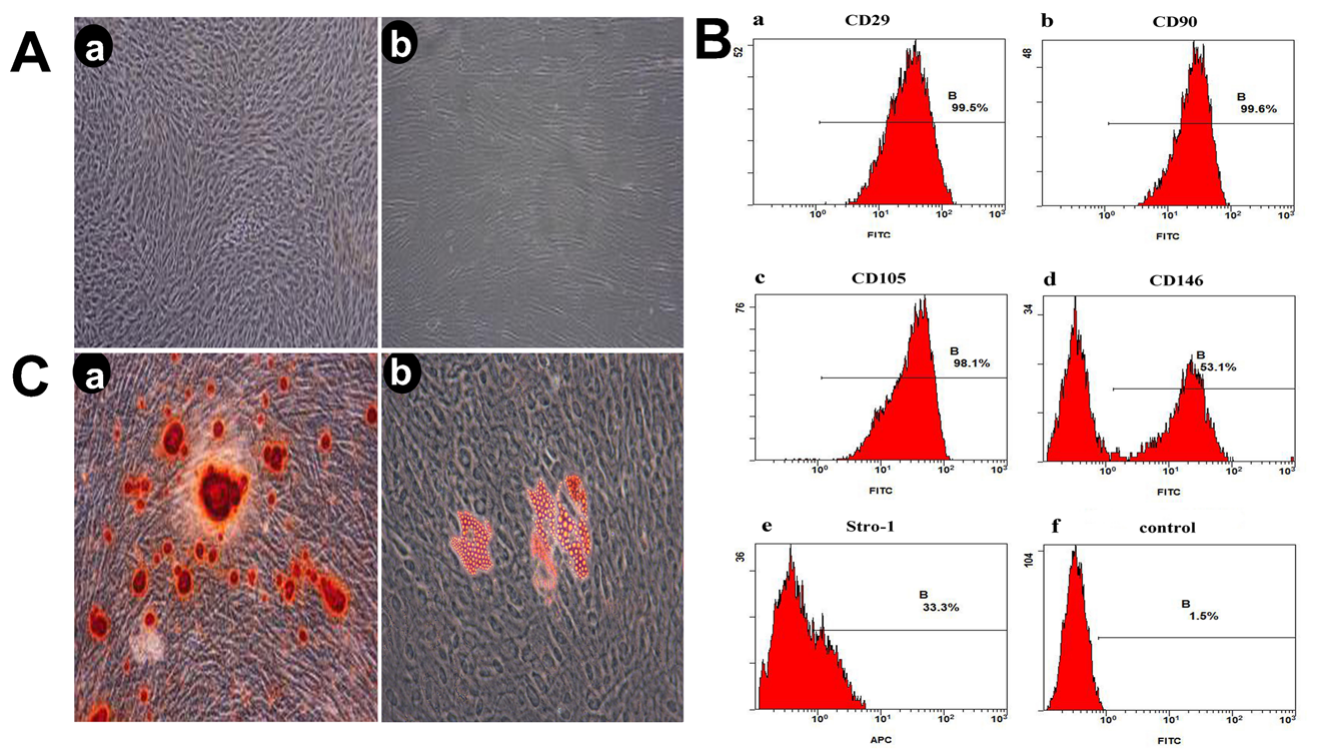
Evaluation of stem cell phenotype. A (Aa) Third-generation cells(x4), (Ab) Third-generation cells (x10); B Detection of cell surface marker characteristic of mesenchymal stem cells on PDLSC Analyses were performed via flow cytometry detecting FITC conjugated monoclonal antibodies for human CD29, CD90, CD105, CD146, Stro-1(Ba, Bb, Bc, Bd, Be), and negative control(Bf); C (Ca) the formation of calcified nodules, (Cb) The formation of lipid droplets.

The tissue block culture method was used to obtain primary cells. After approximately 7 days, cells appeared surrounding the tissue blocks, and reached approximately 80% confluence after 2 weeks. Most of the clonal cells displayed an elongated spindle-like shape with ample cytosol. The logarithmic first-generation cells were seeded at a low density for further purification, and were subjected to normal subculture following protease treatment. Third-generation cells in good condition (Fig 1Aa and 1Ab) were used for further experiments.

### Evaluation of stem cell phenotype

The isolated PDLSC displayed a stem-cell phenotype, and had a comparatively high purity, practically all cells expressed mesenchymal stem cell markers positive for CD29, CD90, CD105, CD146 and STRO-1 (Fig 1B) and negativity for CD14, CD31 and CD45 (S1 Fig).

### Staining for osteogenesis and adipogenesis

After 21 days of osteogenic and adipogenic induction, the cells were stained with alizarin red and oil red O, respectively. Calcification and lipid droplets were clearly visible upon microscopic inspection in the osteogenic and adipogenic induction group, respectively (Fig 1C and 1Cb).

### The effect of a high-glucose microenvironment on the formation of lipid droplets in PDLSC

**Fig 2:**
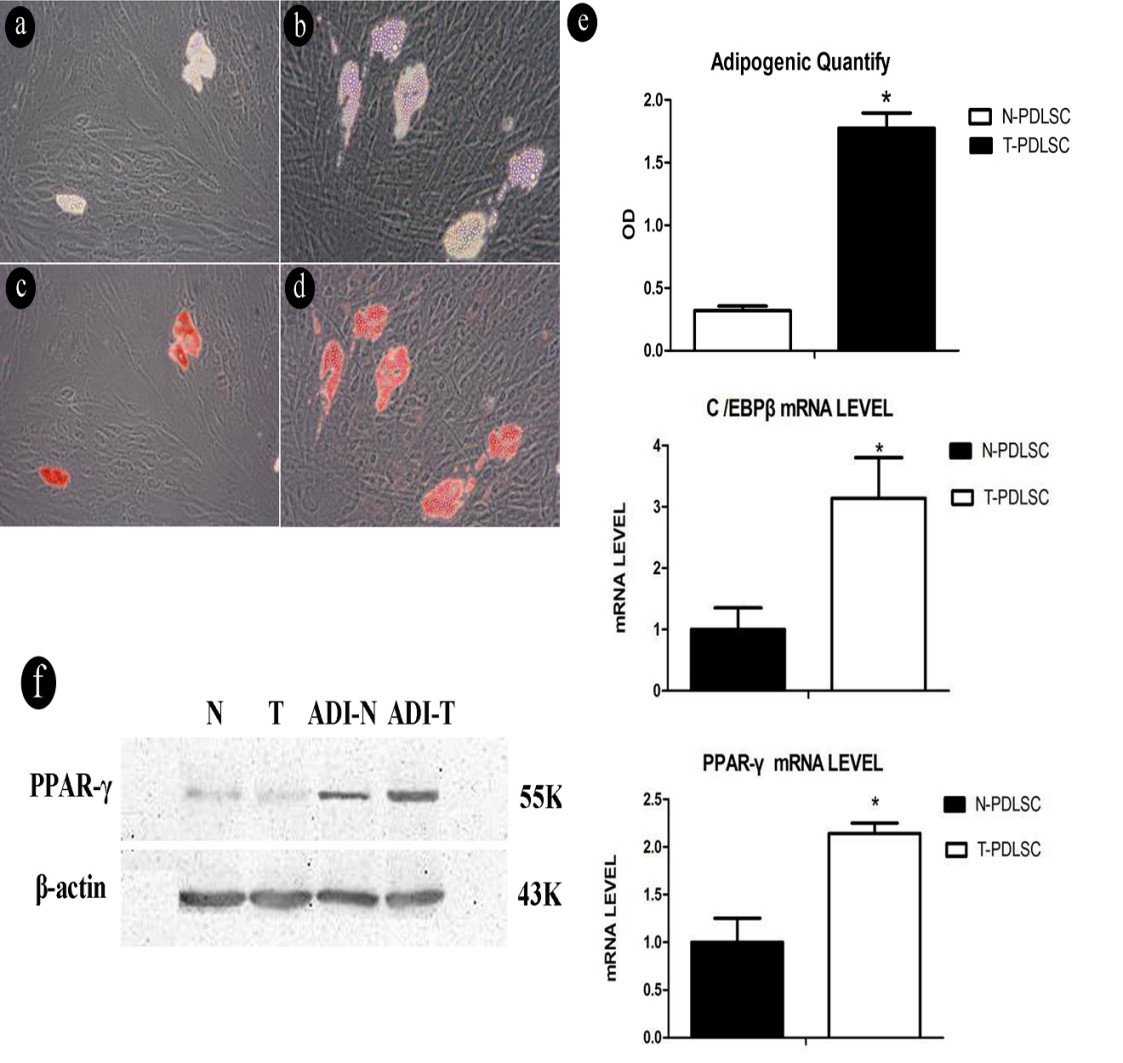
Comparison of lipid droplets formation between normal cell and experimental cell group after adipogenic induction. (2a) Control group before stains, (2b) Experimental group before stains, (2c) Control group after stains, (2d) Experimental group after stains, (2e) Quantitative analysis of isopropanol and the levels of C/EBP beta and PPAR- gamma mRNA were elevated in the experimental group compared with the control group(*p<0.05), (2f) after adipogenic induction, the levels of PPAR-γ protein expression were elevated in the experimental group compared with the control group. N-PDLSC represents normal cell group, and T-PDLSC represents cells were treated with 25 mmol/L glucose.

The cells were randomly separated into two groups, a normal control group comprising periodontal ligament cells grown in DMEM medium with a low glucose concentration (5 mmol/L glucose), and the experimental group cultivated in DMEM medium with a high glucose concentration (25 mmol/L glucose).After simultaneous adipogenic and induction of both groups for 21 days, an increase of intracellular lipid droplets and became visible under the microscope in the experimental group (Fig 2A–2D). The contents of lipid droplets was determined in both groups by staining and subsequent solubilization with isopropanol(Fig 2E).

### Adipogenic differentiation in cells under low and high glucose conditions shows a relationship to C/EBP β and PPAR-γ mRNA expression and protein expression

After simultaneous adipogenic induction in both groups of cells for 7 days, real-time PCR was used to determine the expression of relevant genes. The results demonstrated that the expression of C/EBP β and PPAR-γ mRNA was increased in the experimental group, and the difference was statistically significant (*p<*0.05) (Fig 2E). In addition, the mRNA and protein expression of LPL was also increased in the experimental group (S2 and S3 Figs). After simultaneous adipogenic induction in both groups of cells for 7 days, western blot analysis was conducted to determine the protein expression level of PPAR-γ, and the results showed an increased expression level in the experimental group. (Fig 2F). We have also performed a western-blot assay to detect relative proteins expression of adipogenic differentiation such as adiponectin (S4 Fig), indicating that the low-fat adipogenesis-inducing group was significantly up-regulated, but in the high-glucose-induced group, the expression level was lower than that in the low-glucose-induced group.

### High-glucose microenvironment inhibits the osteogenic differentiation capacity of PDLSCs

**Fig 3:**
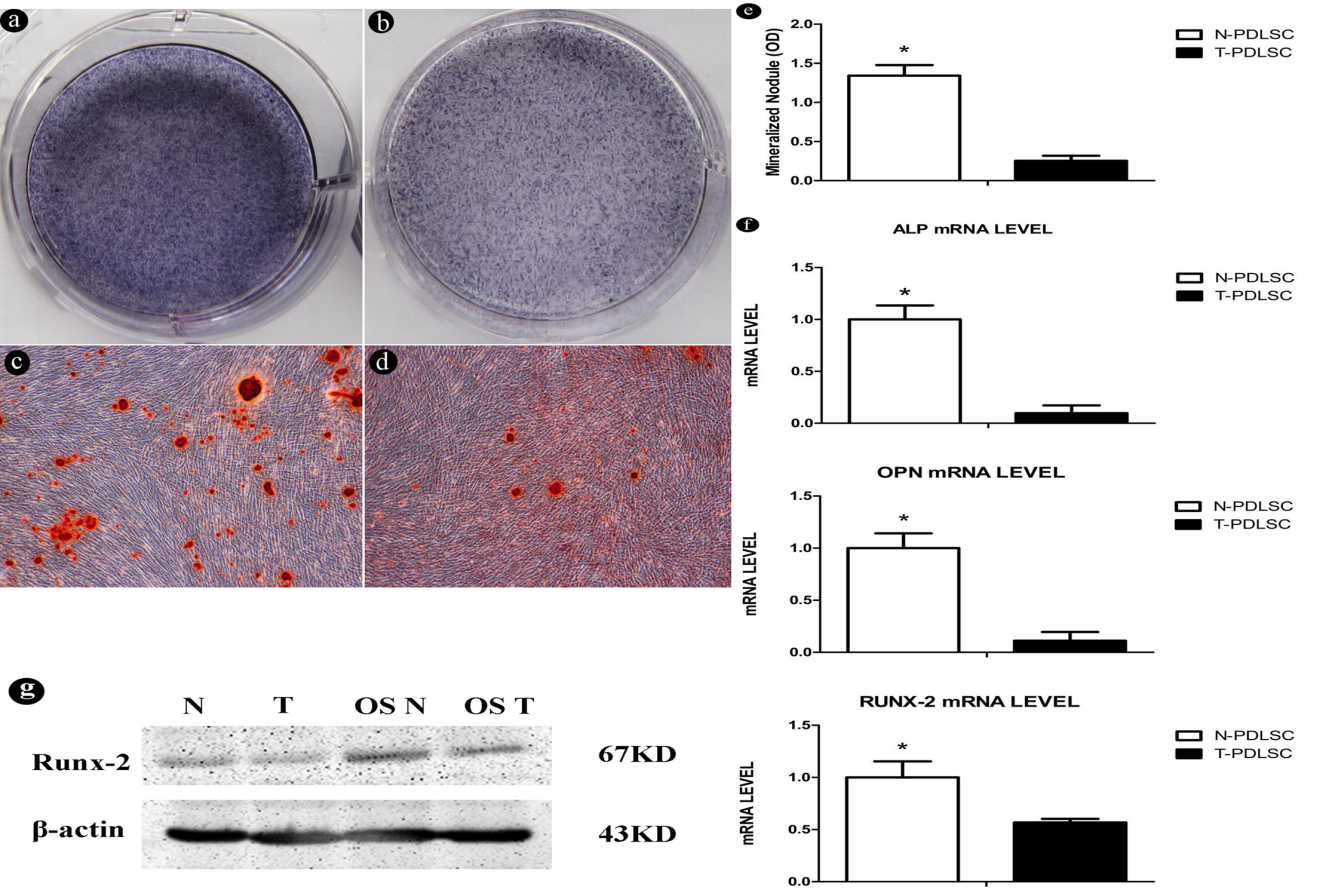
High-glucose inhibits the osteogenic differentiation capacity of PDLSC. (3a) Alkaline phosphatase staining of N-PDLSC group;(3b) Alkaline phosphatase staining of T-PDLSCs group;(3c) Alizarin Red S staining of N-PDLSC group;(3d) Alizarin Red S staining of T-PDLSC group;(3e) Quantification of the amount of Alizarin red staining;(3f) The expression levels of osteoblast marker genes, including ALP, OPN, RUNX-2 in an incubation of 7 days with adipogenic medium.;(3g) The protein levels of Runx-2. N-PDLSC represents normal cell group, and T-PDLSC represents cells were treated with 25 mmol/L glucose.

Stem cells were treated with osteogenic differentiation medium for one week and alkaline phosphatase staining was performed (Fig 3A and 3B). Three weeks later, different nodules appeared in both groups, such as a large number of calcified deposits in the Alizarin Red S staining (Fig 3C), and less deposits in the T-PDLC group (Fig 3D). Similarly, we can find that quantification of Alizarin red staining indicates a significant difference between the two groups (Fig 3E). In addition, on the 21th day, we extracted mRNA from two groups of cultured osteogenic differentiation medium and performed Real Time-PCR to determine the expression of osteoblast marker genes (including ALP, OPN, and RUNX-2) (Fig 3F). Compared with N-PDLC, the marker genes in T-PDLC were reduced. The result of western blotting also showed a decrease in Runx-2 (Fig 3G) and ALP (S5 Fig) protein levels in OST. At the same time, we also performed a western-blot assay to detect related bone protein levels such as OCN and OPN. Our results showed that high glucose inhibited protein expression of OCN (S6 Fig) and OPN (S7 Fig).

### The gene levels of TCF/LEF family

**Fig 4:**
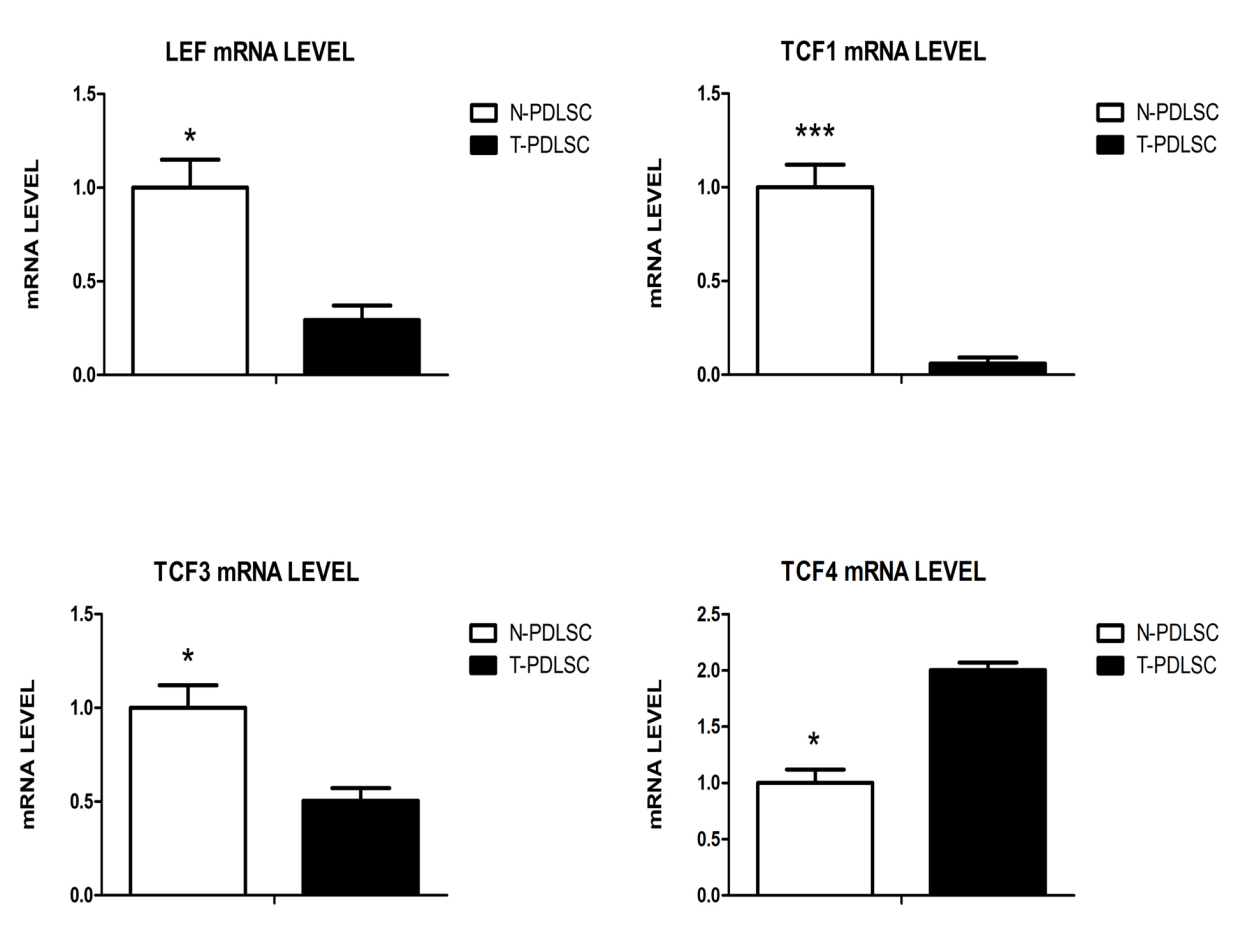
The gene levels of TCF/LEF family.

The results of RT-PCR showed that the expression of LEF, TCF1 and TCF3 mRNA were down-regulated in high glucose group (T-PDLSC) during osteogenic process, with a significant difference (*p*<0.01) compared with normal group (N-PDLSC). Among them, TCF1 was most significantly down-regulated (*p*<0.001), while TCF4 mRNA was up-regulated with a statistically significant difference (*p*<0.01) (Fig 4) in high glucose group (T-PDLSC).

## Discussion

Periodontal tissue damage is one of the most common complications in patients with clinical diabetes[5]. In patients with diabetes, periodontal tissue often appears imbalance of glucose metabolism, reduced protein synthesis, inflammatory cell infiltration, and so on[30, 31]. Studies have shown that PDLSC as seed cells in periodontal tissue can be used to treat periodontal tissue injury[13, 31]. Based on this study, we further understand the biological activity of PDLSC under high glucose environment and the reason of PDLSC have a limit for periodontal tissue repair due to its selective adipogenic differentiation.

The results showed that: (1) high glucose can inhibit the osteogenic differentiation of PDLSC; (2) high glucose can promote the formation of lipid droplets and increase the adipogenic differentiation of PDLSC; (3) High glucose can promote the lipidation process by increasing the expression of C/EBP β and PPAR-γ mRNA.

C/EBP β is an important member of the C/EBP transcription factor family, which regulates the transcription of target genes that take part in cell proliferation, differentiation, tumorigenesis, and similar important biological activities[32]. C/EBP β is also an early transcription factor that induces the formation of adipocytes. It can activate the PPAR-γ gene, whereby PPAR-γ is involved in the regulation of lipid metabolism and immunity, and can control the expression of many kinds of genes involved in metabolic pathways of lipids, and activate adipocyte-specific genes, including those involved in fatty acid transport, cellular uptake and intracellular fatty acid synthesis, as well as degradation and storage[33, 34]. The results of this study also show that C/EBP β and PPAR-γ are both upregulated during the adipogenic differentiation process of human PDLSC under the influence of a high-glucose microenvironment. This implies that the C/EBP β and PPAR-γ genes are activated, and therefore in turn promote adipogenic differentiation.

We have also observed that the number of lipid droplets gradually increased during the adipogenic differentiation process of human PDLSC under a high-glucose microenvironment. Furthermore, an investigation of gene expression levels revealed that C/EBP β and PPAR-γ were expressed in large amounts, which shows that C/EBP β exerted its function. During the early stages of the differentiation process of 3T3-L1 adipocytes, C/EBP β can activate PPAR-γ through multiple pathways related to lipid metabolism, whereby C/EBP β transactivates a large number of genes related to the cell cycle, including G9a, which promotes mitotic clonal expansion (MCE). MCE is the final necessary step of adipogenic differentiation, and during the late stages of the differentiation of 3T3-L1 adipocytes, C/EBP independently activates the unfolded protein response (UPR) via the transcription of Xbp1 and Atg4b, and can thereby inhibit the expression of the adipocyte differentiation gene wnt10b[35, 36]. Under the influence of this series of signals, the PPAR-γ gene is finally upregulated, which induces final adipocyte differentiation. In this research, we demonstrated that a high-glucose microenvironment can promote the adipogenic differentiation of human PDLSC, thereby inducing the adipogenic development of periodontal tissue, which influences the tissue’s regeneration and survival. In addition, we extracted mRNAs cultured in osteogenic differentiation medium for 7 days. We found that the expression of osteoblast markers including ALP, OCN, and RUNX-2 genes was significantly reduced in T-PDLC. Western blot analysis also showed that Runx-2 protein levels were also reduced in T-PDLC. According to this line of reasoning, it is clear why the periodontal tissue of clinical diabetic patients is prone to damage, and also recovers poorly.

Studies have shown that Wnt/β-catenin signaling pathway is considered as one of the signaling pathways that play a key role in the proliferation and differentiation of stem cells, and plays an important role in the formation of bone[37]. Furthermore, the four transcription factor family LEF/TCF (TCF1, TCF3, TCF4, LEF) play a molecular switch in the Wnt/β-catenin signaling pathway[38]. Our results showed that under high glucose treatment, the expression levels of TCF1, TCF3, and LEF genes in stem cells were significantly reduced, whereas TCF4 was significantly elevated. This result implies that our high glucose promotes the adipogenic process of stem cells rather than the osteogenic process, which can only initially explain the reasons for the difficult recovery of periodontal tissue defects in diabetic patients. However, there are still deficiencies in our experiments, such as the use of gene silencing methods to further confirm the regulation mechanism of high glucose on these four transcription factors. We will solve this problem in the future, which is of reference for us to find therapeutic strategies.

However, this study was not able to uncover the molecular pathways through which the advanced glycation end products influenced the expression of PPAR-γ, or which downstream genes were influenced by the change of PPAR-γ expression. This important question will be the subject of our future research.

## Conclusion

In conclusion, our results indicate that high glucose can promote the adipogenic differentiation and inhibit osteogenic differentiation in PDLSC by regulating mRNA and protein expression of C/EBP β, PPAR-γ. These results explain the reasons for the difficulty of periodontal healing in diabetic patients and at the same time lays a foundation for the development of drugs for periodontal healing.

## Supporting information

S1 Fig Flow cytometer analysis negativity for CD14(A), CD31(B) and CD45(C).
(TIF)

S2 Fig The mRNA expression of LPL. N-PDLSC represents normal cell group, and T-PDLSC represents cells were treated with 25 mmol/L glucose.
(TIF)

S3 Fig The original uncropped protein expression of LPL. L (low glucose group), H (high glucose group), ADI-L (low sugar adipogenesis induction group), ADI-H (high glucose adipogenesis induction group).
(TIF)

S4 Fig The original uncropped protein expression of Adiponectin. L (low glucose group), H (high glucose group), ADI-L (low sugar adipogenesis induction group), ADI-H (high glucose adipogenesis induction group).
(TIF)

S5 Fig **The original uncropped protein expression of ALP and RUNX-2 (A) and β-actin (B)**. L (low glucose group), H (high glucose group), OSL (low glucose osteogenic induction group), OSH (high glucose osteogenic induction group).
(TIF)

S6 Fig The original uncropped protein expression of OCN. L (low glucose group), H (high glucose group), OSL (low glucose osteogenic induction group), OSH (high glucose osteogenic induction group).
(TIF)

S7 Fig The original uncropped protein expression of OPN. L (low glucose group), H (high glucose group), OSL (low glucose osteogenic induction group), OSH (high glucose osteogenic induction group).
(TIF)
