# Supplementary figures and images for: Selective adipogenic differentiation of human periodontal ligament stem cells stimulated with high doses of glucose
Source: PLoS One. 2018 Jul 6;13(7):e0199603. doi: 10.1371/journal.pone.0199603 (PMC6034828; doi:10.1371/journal.pone.0199603)

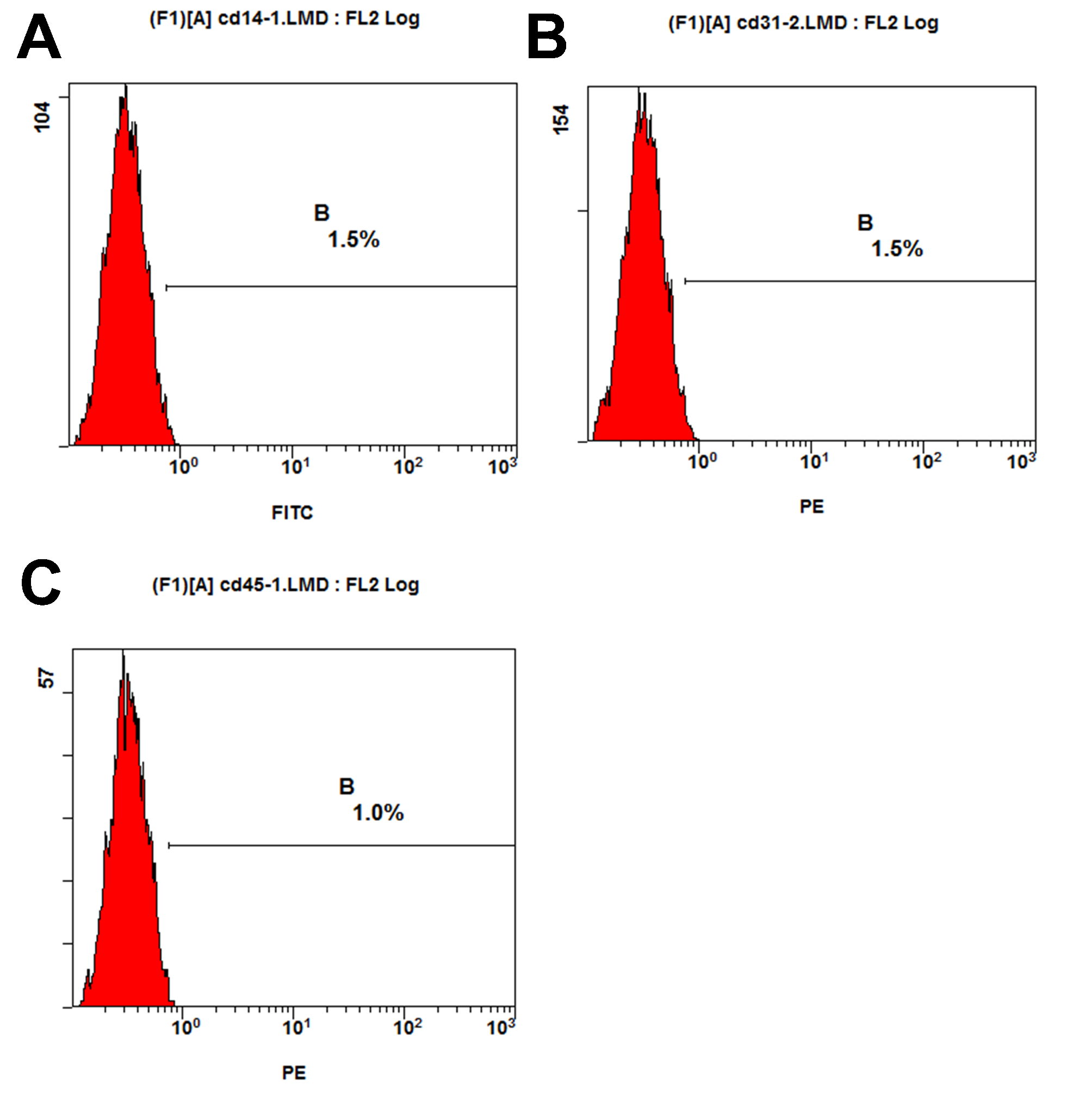

Supplement: S1 Fig — Flow cytometer analysis negativity for CD14(A), CD31(B) and CD45(C). (TIF) [file pone.0199603.s001.tif]

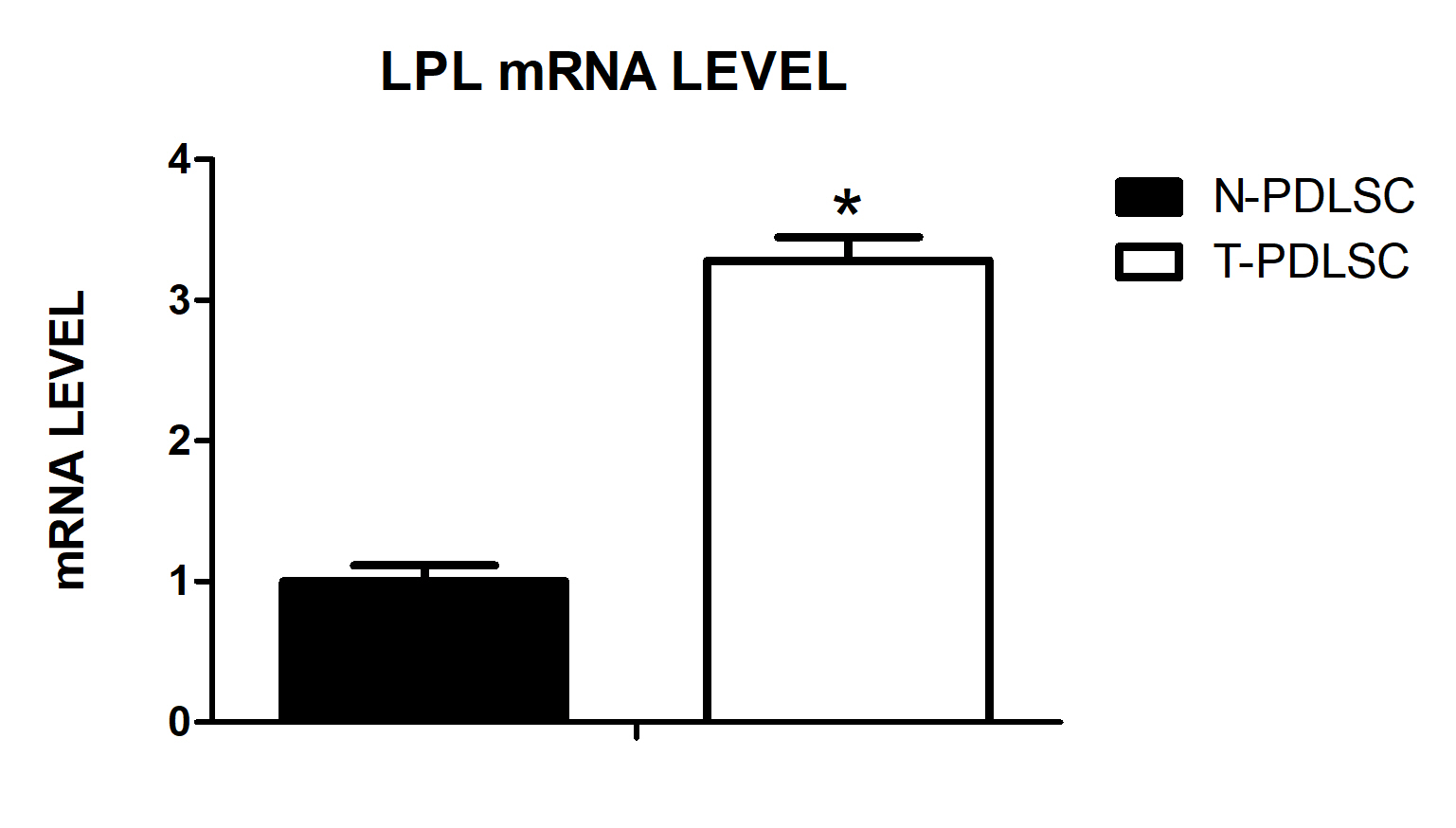

Supplement: S2 Fig — N-PDLSC represents normal cell group, and T-PDLSC represents cells were treated with 25 mmol/L glucose. (TIF) [file pone.0199603.s002.tif]

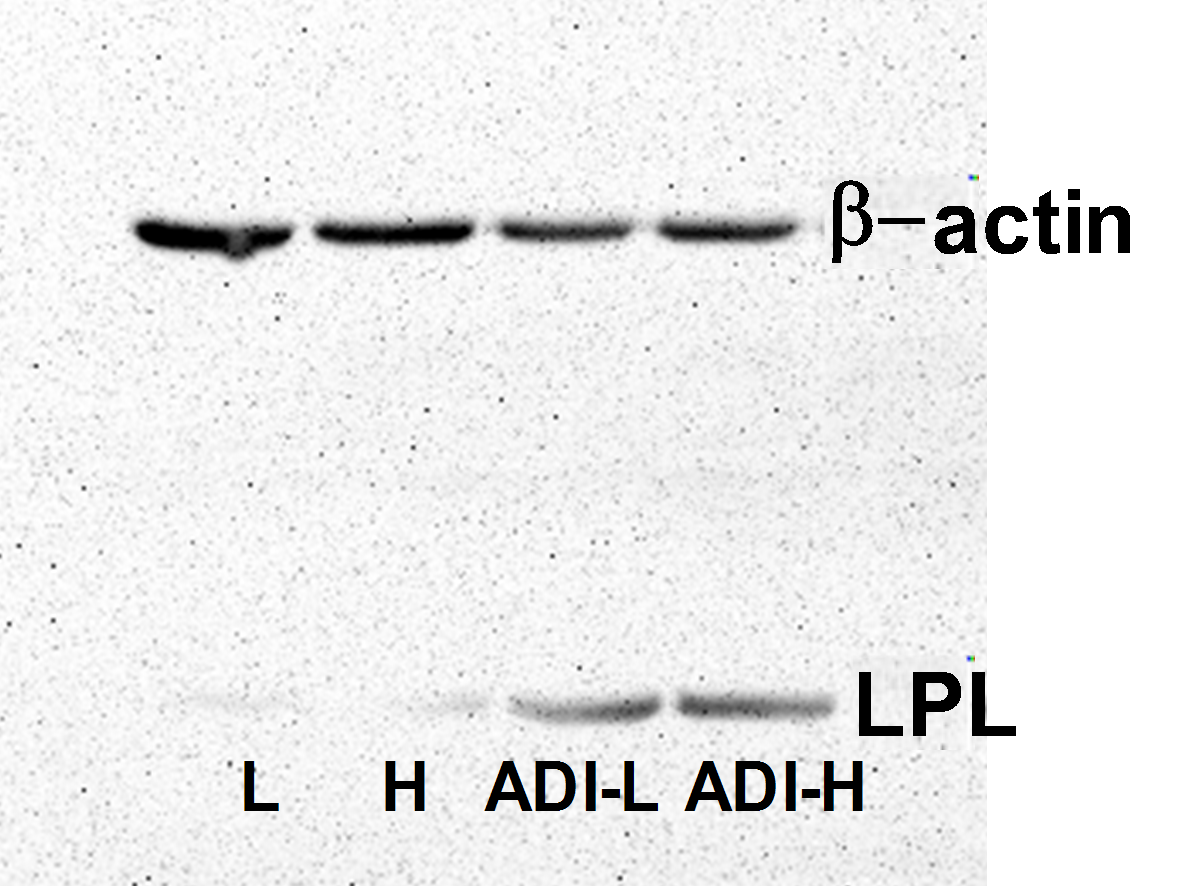

Supplement: S3 Fig — L (low glucose group), H (high glucose group), ADI-L (low sugar adipogenesis induction group), ADI-H (high glucose adipogenesis induction group). (TIF) [file pone.0199603.s003.tif]

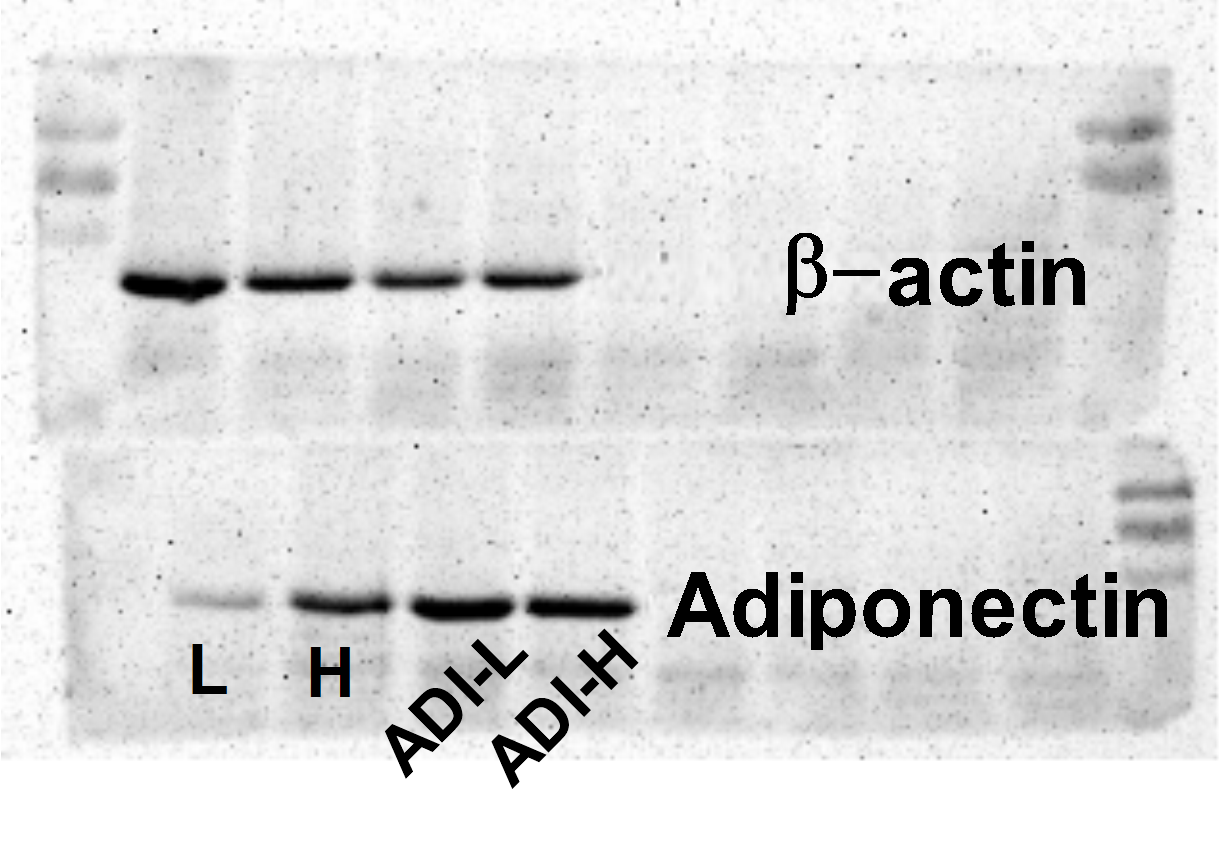

Supplement: S4 Fig — L (low glucose group), H (high glucose group), ADI-L (low sugar adipogenesis induction group), ADI-H (high glucose adipogenesis induction group). (TIF) [file pone.0199603.s004.tif]

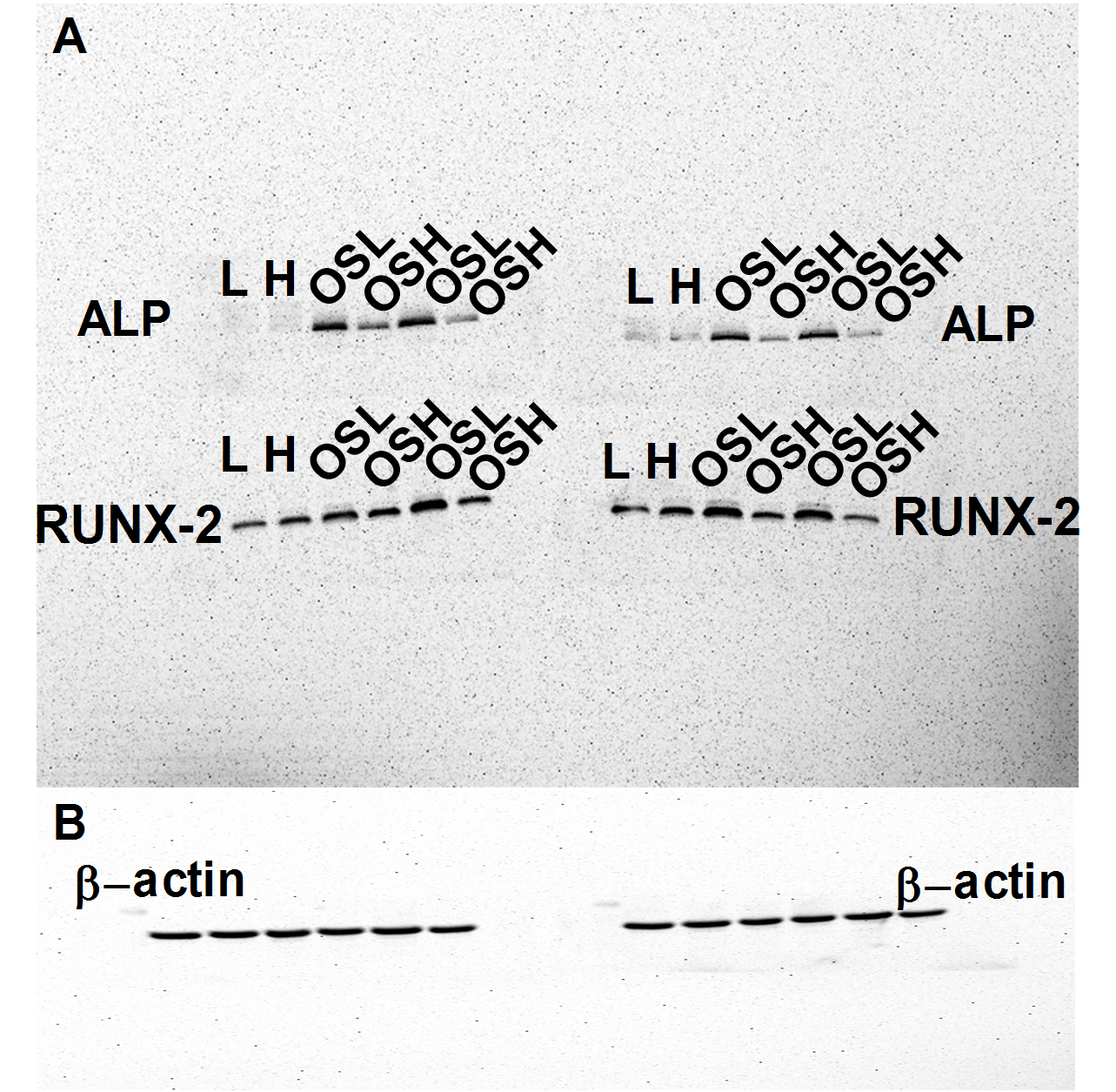

Supplement: S5 Fig — The original uncropped protein expression of ALP and RUNX-2 (A) and β-actin (B). L (low glucose group), H (high glucose group), OSL (low glucose osteogenic induction group), OSH (high glucose osteogenic induction group). (TIF) [file pone.0199603.s005.tif]

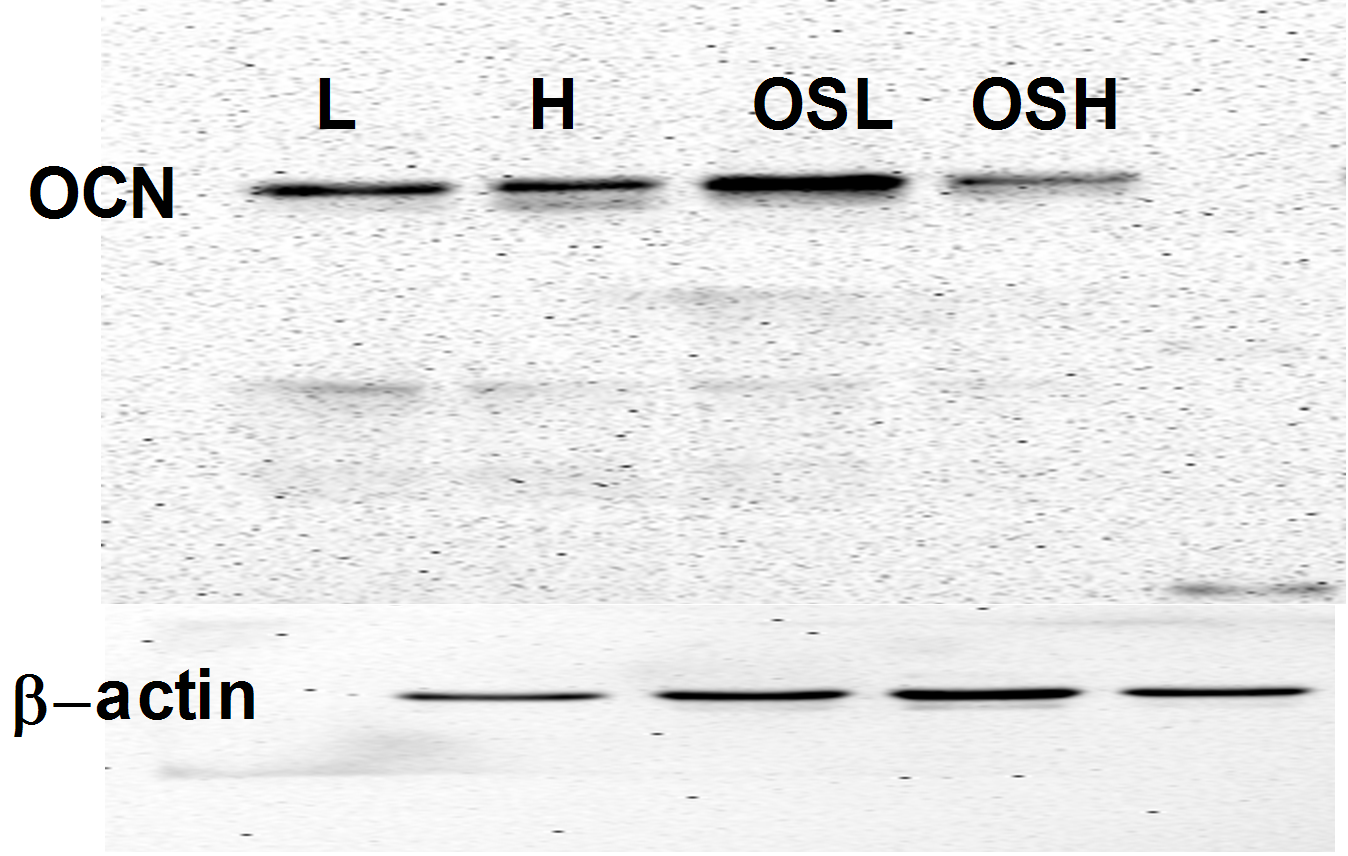

Supplement: S6 Fig — L (low glucose group), H (high glucose group), OSL (low glucose osteogenic induction group), OSH (high glucose osteogenic induction group). (TIF) [file pone.0199603.s006.tif]

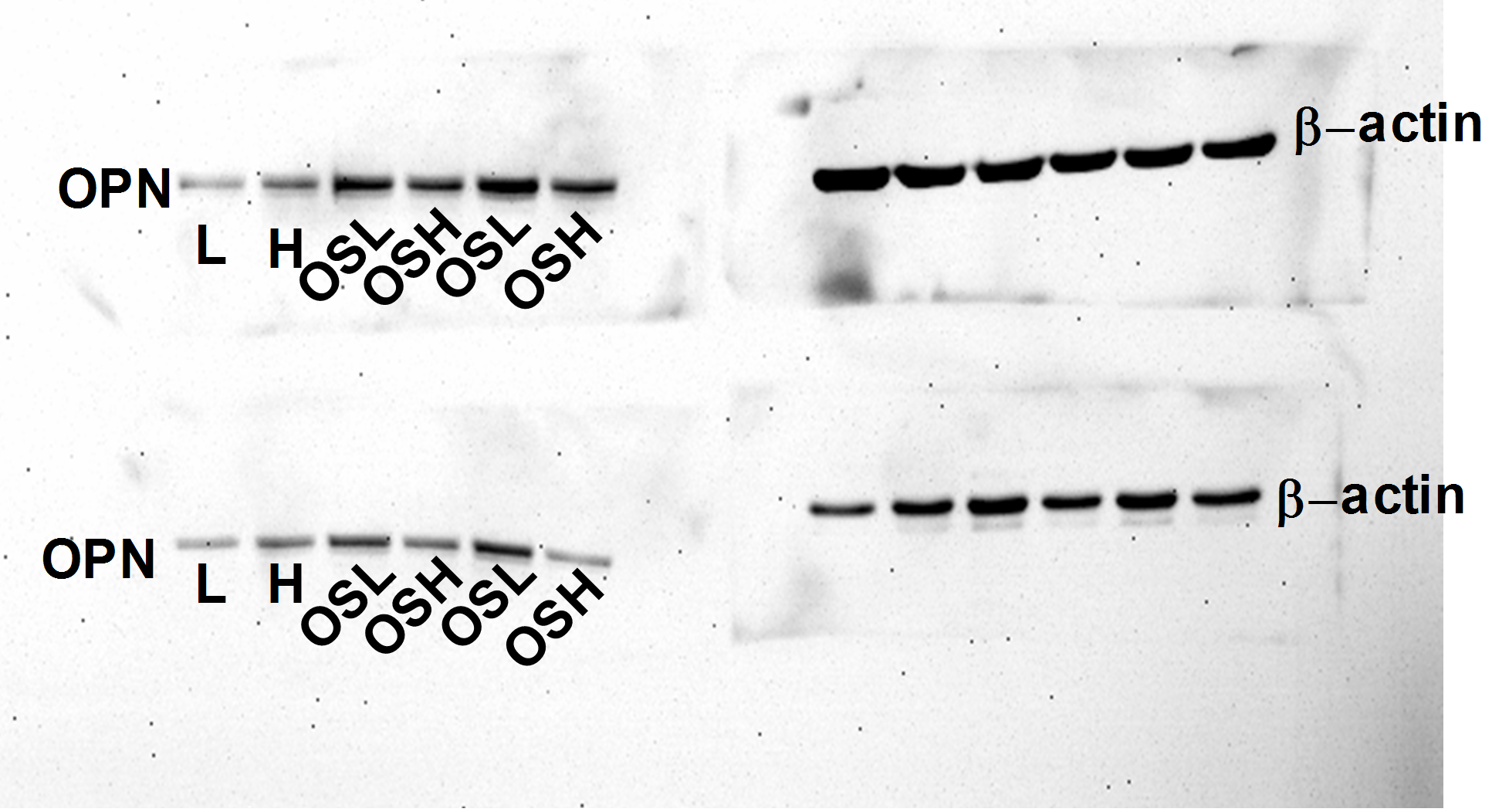

Supplement: S7 Fig — L (low glucose group), H (high glucose group), OSL (low glucose osteogenic induction group), OSH (high glucose osteogenic induction group). (TIF) [file pone.0199603.s007.tif]
